# Gold Foil

**Published:** 1867-05

**Authors:** 


### ARTICLE II.

### *Gold Foil.*

THE manufacture of the Gold Foil used for filling teeth, has attained a greater perfection in this country than in any other, and the process is one of peculiar interest to the dental profession.

We are indebted to Mr. W. R. Abbey, of Charles Abbey and Sons, the oldest Gold Foil manufacturers in America, for the material of which this article is composed, it being a portion of one prepared for the new edition of Harris' Medical and Dental Dictionary.

Gold Foil as used by dentists for filling teeth, is gold hammered into a thin leaf, but finer in quality and of much greater thickness than the article ordinarily known as Gold Leaf. As at present supplied to the dentist, it is divided into Soft or Plain Gold Foil, and Adhesive Gold Foil, the appreciable difference between them being that the latter possesses the quality of adhering or welding together with much less pressure, when freshly prepared, than the former.

The thickness of the individual leaves or sheets is, or should be indicated by the expression of the weight in grains, of each sheet. Thus a sheet of No. 4 should weigh four grains, No. 5 five grains, and so on; consequently a Troy ounce of No. 4 contains 120 sheets, while the same weight of No. 6 will contain only 80 sheets. The numbers most in use are 4, 5, 6 and 8, though occasionally thicker numbers are called for.

When properly prepared, Gold Foil is made from absolutely *pure* gold, and particular attention given to the annealing process by the manufacturer; this latter is of as much importance as the former. There are various methods of freeing Gold from foreign matter or alloy, but the most effectual and certain method by which gold can be made absolutely pure is by dissolving it in Aqua Regia (Royal

Water,) a mixture of nitric and muriatic acids in proportion of one part of the former, to four of the latter. The bullion to be refined (composed say ot gold, silver and copper,) prepared by graining or passing through the rolls, is put into a glass matrass and a suitable quantity of the Aqua Regia poured on it, and then submitted to a heat in a water or sand bath. The gold and the copper are dissolved and remain in solution, while the silver is precipitated to the bottom of the mattrass as a chloride in a greyish white powder. The solution must be carefully decanted from the chloride into a solution of protosulphate of iron, at the bottom of which, after a short interval, the gold will be found precipitated in form of a reddish brown powder. This precipitate must be well digested in muriatic acid, then in boiling water, and after drying may be melted with a little borax.

The whole operation is a very delicate one, requiring considerable experience, and the exercise of great patience, care and attention, to ensure success.

The gold is cast into ingots about one inch wide, and portions of it (varying in weight according to the number intended to be made) are cut off and passed between fine steel rolls, until the proper thickness is reached, which for number 4 is when a piece of the "ribbon" one inch square will weigh about five grains.

Two hundred of these inch square pieces of gold are "filled" into the centre of a four inch square packet composed of pieces of vellum, or of a peculiar paper, a square of gold and a piece of the vellum or paper, alternating all the way through.

The packet which is technically called a "cutch" is then tightly encased on all sides by strong parchment casings, and is ready for "beating." The hammers used weigh from 12 to 16 pounds, and are wielded with one hand, the other being employed in regularly turning the cutch around and over, so as to bring every part of it equally under the hammer.

The beating is commenced upon the centre of the cutch where the squares of gold are piled, but as the squares enlarge by the force of the blows, the direction of the hammering is moved outward apace, the skill of the workman being proved by his ability to keep the enlarging gold in the cutch as nearly square as when started. The beating is continued until the edges of the gold are driven out beyond the edges of the cutch, when it is carefully scraped off and weighed from time to time, until the proper quantity has been taken off. The sheets of foil are then laid out from the cutch, the rough edges trimmed smooth and even, and they are ready for the process of softening or annealing. This is an important process and each manufacturer has his own method of doing it, the details of which are seldom made known. The general principle is, that by exposure to heat, the soft, kid-like quality of absolutely pure gold may be restored to sheets of foil that have been rendered hard, harsh, and unyielding by the hammering they have been subjected to. Some manufacturers do it by placing a sheet of the foil upon a wire grating, and holding it over a fire, or spirit lamp; others heat a plate of stone, and lay the gold upon it, whilst others again place it directly on a charcoal fire. After annealing, the foil is placed in books preparatory to exposure for sale.

